# Epigenetics and Triplet-Repeat Neurological Diseases

**DOI:** 10.3389/fneur.2015.00262

**Published:** 2015-12-21

**Authors:** Sathiji Nageshwaran, Richard Festenstein

**Affiliations:** ^1^Division of Brain Sciences and MRC Clinical Sciences Centre, Faculty of Medicine, Imperial College London, Hammersmith Hospital Campus, London, UK

**Keywords:** epigenetics, neurology, triplet repeat, HDAC, Friedreich’s ataxia, neurogenetics, FRDA, heterochromatin

## Abstract

The term “junk DNA” has been reconsidered following the delineation of the functional significance of repetitive DNA regions. Typically associated with centromeres and telomeres, DNA repeats are found in nearly all organisms throughout their genomes. Repetitive regions are frequently heterochromatinized resulting in silencing of intrinsic and nearby genes. However, this is not a uniform rule, with several genes known to require such an environment to permit transcription. Repetitive regions frequently exist as dinucleotide, trinucleotide, and tetranucleotide repeats. The association between repetitive regions and disease was emphasized following the discovery of abnormal trinucleotide repeats underlying spinal and bulbar muscular atrophy (Kennedy’s disease) and fragile X syndrome of mental retardation (FRAXA) in 1991. In this review, we provide a brief overview of epigenetic mechanisms and then focus on several diseases caused by DNA triplet-repeat expansions, which exhibit diverse epigenetic effects. It is clear that the emerging field of epigenetics is already generating novel potential therapeutic avenues for this group of largely incurable diseases.

## Epigenetic Mechanisms

Transcription is tightly regulated in all cells. This is done in part through a number of epigenetic mechanisms, namely: heterochromatin formation and associated histone modifications, DNA methylation, antisense transcription, and formation of complex DNA structures, such as DNA–RNA hybrids, RNA loops, and DNA triplexes (1). Working alone or in unison, these mechanisms allow for a dynamic state of gene expression. These mechanisms have been implicated in a variety of disease states, with the enticing potential that they may be amenable to therapeutic intervention.

DNA methylation is the most studied epigenetic modification and is heavily implicated in many oncological disorders. Methylation in adult somatic cells is primarily found on CpG nucleotides and this modification is maintained through cell division (2). DNA methylation is thought to have developed as a means to silence viral genes that have been integrated into a host organism’s genome over time.

**Figure 1:**
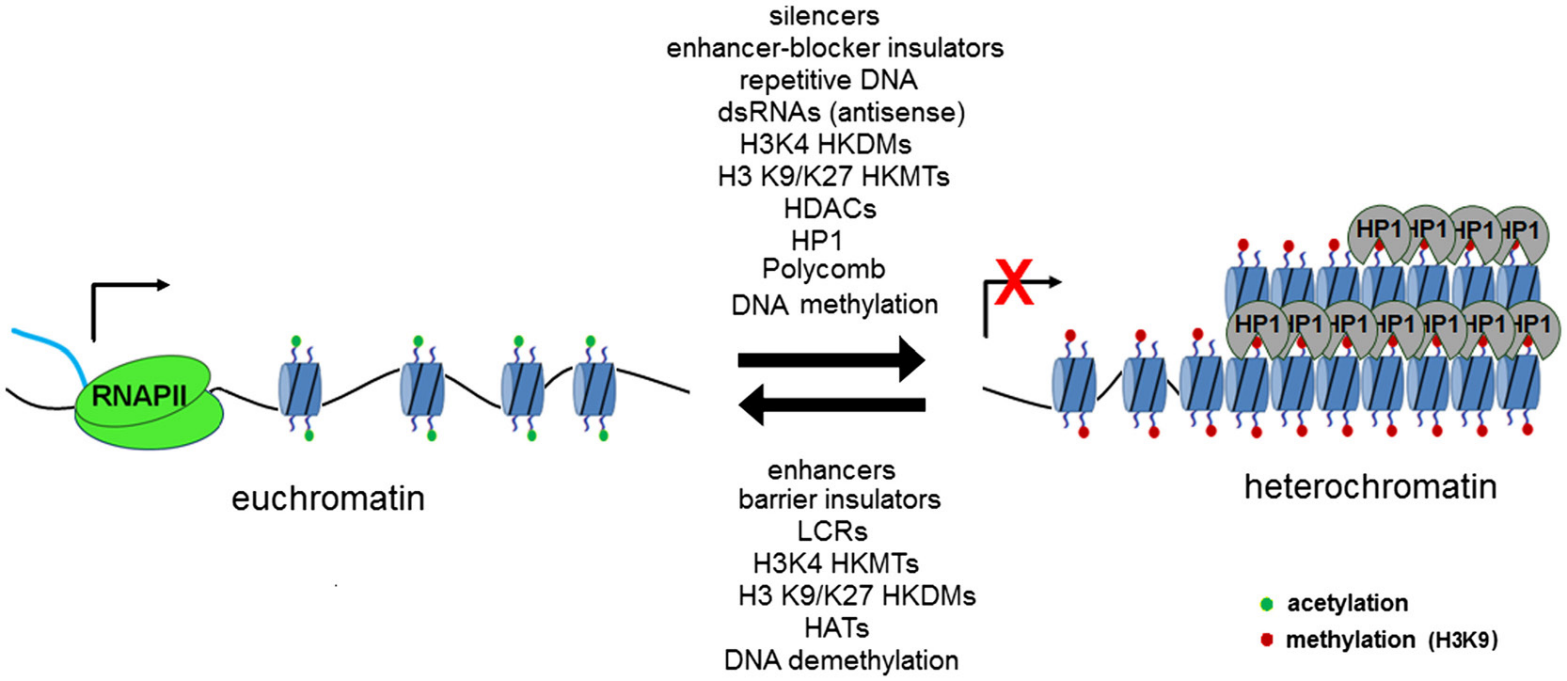
**Transcriptionally permissive euchromatin may be remodeled in a reversible fashion through the action of a number of chromatin-modifying elements, which act to methylate lysine residues in position 9 (SUV39H associated with constitutive heterochromatin) and position 27 (Polycomb repressor complex associated with facultative heterochromatin)**. Regions of repetitive DNA have been shown to promote the formation of heterochromatin. Figure used with permission from Yandim PhD thesis 2012, Imperial College London.

DNA is transcribed by RNA polymerase II along the template strand in the 5′–3′ direction. This results in the formation of primary or nascent RNA, which is subsequently spliced to remove introns and polyadenylated to form mRNA. Initially described in yeast, antisense transcription (transcription of the antisense strand in the 3′–5′ direction) is now known to be important in the regulation of gene expression through mechanisms, including RNA interference and the formation of RNA–DNA complexes (3). The most widely recognized antisense transcript implicated in gene regulation is Xist, which is known to cause X chromosome inactivation (4). The random action of Xist on one of a cell’s X chromosomes results in DNA methylation, the addition of silencing histone modifications and subsequently widespread heterochromatinization (3). DNA is packaged within the nucleus into a compact structure known as chromatin. Nucleosomes form the functional unit of chromatin, whereby a 147 base pair length strand of DNA is wrapped around a histone octamer core. Chromatin can be further packaged into dense regions known as heterochromatin, which are often transcriptionally silenced (5). The mechanisms that dictate whether a region of DNA undergoes heterochromatinization are still being elucidated, with a dynamic state being implicated by the reversible nature of “silencing marks” (histone lysine methylation or acetylation) associated with heterochromatin and the dosage-dependent effects of various heterochromatin modifiers, such as heterochromatin protein 1 (HP1) and suppressor of variegation 3-9 (SUV39H) (6). Transcription factors have also been shown to regulate silencing of repeat regions, with sequence-specific binding sites for several transcription factors (e.g., Pax3) located in repetitive DNA. Disruption of these binding sites may result in a reduction in heterochromatic marks (e.g., H3K9me3) and release from silencing of heterochromatin (7). Coupling of repeat length and transcription factor dosage may provide an explanation for the close link in several disorders between repeat length, disease onset, and severity. This interplay between positive and negative factors (Figure 1) regulating heterochromatin formation and/or transcription has allowed novel treatments to be trialed for a number of neurological disorders with the aim of restoring expression of pathologically silenced genes with the promise that such an approach might lead to disease-modifying therapies for several as yet incurable diseases (8, 9).

DNA is most commonly found in a double helical structure, also known as B-DNA, and is the result of complementary base pairing between nucleotides. The sequence of nucleotides plays an important role in the structure adopted by DNA. Non-B DNA structures, such as triplexes and tetraplexes, and DNA–RNA hybrids are implicated in the pathogenesis of several trinucleotide repeat disorders through their effect on transcription, DNA replication, and genomic stability (10).

## Triplet-Repeat Diseases and Epigenetics

### Friedreich’s Ataxia

Friedreich’s ataxia (FRDA) is the commonest inherited ataxia, with a prevalence between 1 in 20,000 and 1 in 50,000 among caucasians (11, 12). Over 98% of cases are the result of a (GAA)n triplet-repeat expansion within intron 1 of the *frataxin* (*FXN*) gene, the rest being the result of compound heterozygosity with an expansion on one allele and a point mutation or insertion on the other (13). Both result in gene silencing and a downregulation of frataxin protein, which causes FRDA. Frataxin levels can be used to differentiate unaffected individuals, carriers, and FRDA patients (14). There is no evidence that frataxin itself is dysfunctional in FRDA, with no defect in mRNA half-life or splicing between patients and unaffected individuals (1, 15–17). Frataxin is a mitochondrial protein important in iron-sulfur cluster biogenesis and intracellular iron homeostasis. Various mechanisms have been put forward for the reduction in expression, including adoption of abnormal secondary DNA structures by long GAA tracts (DNA triplexes and R-loops), problems in transcription initiation and/or elongation and more recently greatest attention has focused on epigenetic gene silencing (16). Epigenetics broadly describes processes that alter gene expression without a change in nucleotide sequence (1, 18). Epigenetic silencing of the *FXN* gene has been shown *in vivo*, and a number of therapeutic agents have been shown to “switch” the *FXN* gene back on and are in early stage clinical trials (17). Other clinical treatments have aimed at the downstream effects of frataxin deficiency, such as boosting mitochondrial function (e.g., coenzyme q10 and idebenone) and iron chelation (19–23).

**Figure 2:**
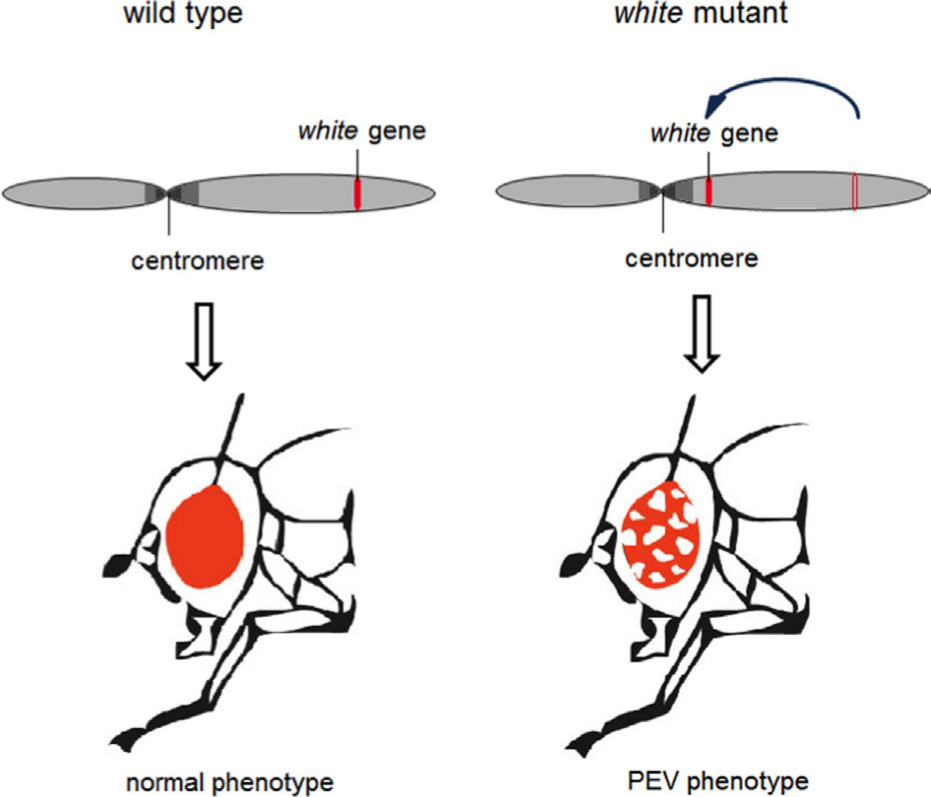
**Radiation-induced translocation of the white gene, which is responsible for the fly’s red eye color, near to a region of heterochromatin results in silencing of the gene in a proportion of cells that is clonally stable**. Figure used with permission from Yandim PhD thesis 2012, Imperial College London.

The first evidence for the epigenetic silencing mechanism behind FRDA came from experiments in mice where a human CD2 (hCD2) reporter transgene was linked to triplet-repeat expansions (GAA a or CTG) (24). Here, insertion of a hCD2 transgene without the associated repeats but near centromeric repetitive heterochromatic regions resulted in variegated silencing that resembled the archetypal epigenetic silencing known as position effect variegation (PEV) (24). PEV was first shown in *Drosophila* when the *white* gene (which encodes red eye color) was translocated close to pericentromeric repeats resulting in silencing in a proportion of cells, which is clonally stable. It has been instrumental in unraveling the molecular basis for heterochromatin formation and stochastic silencing of the affected gene (Figure 2) (25).

**Figure 3:**
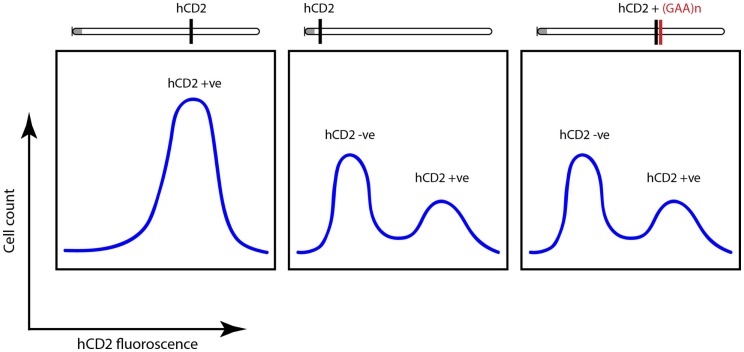
**Fluorescence-activated cell sorting (FACS) plots highlighting variegated silencing of hCD2 reporter transgene when located near centromeric heterochromatin (gray dot)**. When attached to GAA-repeat expansion, a similar silencing was noted independent of the location of the transgene. Adapted from Yandim et al. (13).

Genetic screens identified powerful suppressors of variegation (silencing), which were components of the silencing machinery. These included a highly conserved histone methyltransferase, Suvar 3-9 which methylates histone H3 on the N-terminal tail that protrudes from the nucleosome, helping to establish the notion of an “epigenetic” or “histone code”(26) whereby heterochromatin is “labeled” by histone H3 lysine 9 (K9) trimethylation (me3) and this “label” is recognized and bound by the product of another powerful genetic modifier of PEV – heterochromatin protein 1 (Suvar205) (26). These findings established a possible mechanism for heterochromatin formation and spreading. That these mechanisms are conserved in mammals was established using the hCD2 transgenic model for PEV where it was also shown that expressing transgenes carry acetylation marks on their histones and non-expressing transgenes from the same mice bear H3K9me3 (24, 27). When a pathological GAA tract was linked to this transgene, it triggered variegated expression, which was independent of the location of the construct along the chromosome (Figure 3) (24).

Subsequent work has supported aspects of this mechanism in FRDA with the identification of histone modifications frequently associated with constitutive (H3K9me3) and facultative (H3K27me3) heterochromatin being found flanking the GAA repeat as well as a reduction in acetylated H3 and H4 (marks associated with active chromatin) (12). The coexistence of both H3K9me3 and H3K27me3 is unusual, with the latter mark the result of methylation by another methyltransferase (enhancer of zeste – EZH2) (28). H3K27me3 is recognized by a protein called Polycomb which is part of the Polycomb Repressor Complex (29). A number of factors are known to regulate a gene’s epigenetic landscape, such that an alteration in the dosage of “positive” or “negative” factors can tip the scale in favor of gene expression or repression, respectively (6). To this end, histone deacetylase inhibitors (HDACi) have shown promising results in promoting frataxin expression in animal and human studies (30–33). Histone deacetylases (HDACs) are a group of enzymes that act by removing acetylation marks from histones, which subsequently allow for their methylation by histone lysine methyl transferases (HMTs) (13). Studies in mice have shown that these compounds are able to significantly upregulate frataxin expression in disease-specific tissues (heart, brain, cerebellum, and dorsal root ganglia) (30). The earliest work on the role of HDACi in FRDA was completed by Gottesfeld and colleagues who found that BML-210 and its synthesized derivatives (pimelic diphenylamides) HDACis were able to increase frataxin expression in cellular models and primary lymphocytes (33). Compound 109, synthesized for greater specificity for HDAC3, has since been taken into an early phase clinical study where it increased frataxin expression in peripheral blood mononuclear cells (PBMCs) from patients and reduced H3K9me3 (31–38).

Another such compound is nicotinamide, vitamin B3, a class III HDACi. Nicotinamide treatment of FRDA mice and primary lymphocytes in culture resulted in a permissive environment for transcription, as suggested by an increase in euchromatic histone marks and a reduction in heterochromatin marks, increased frataxin production and correction of 67% of genes known to be dysregulated in FRDA (17). The higher-order architecture of the abnormal *FXN* gene was shown by chromosome conformation capture (3C) to be remodeled following nicotinamide treatment with a reduction in interaction frequency of regions flanking the GAA repeat containing anchor fragment and increased DNAse I accessibility, both implying a more open chromatin structure (17). In the first trial of an epigenetic therapy for a disorder outside cancer, nicotinamide was safely used in patients with FRDA (19). In this study, nicotinamide was administered daily at high dose for 8 weeks with an increase in frataxin protein to levels seen in asymptomatic carriers, measured from PBMCs. Although the short duration of the study would not allow for changes in disease severity to be measured by recognized clinical scales [e.g., Scale for the Assessment and Rating of Ataxia (SARA)], a suggestion of improvement was noted in patient’s activities of daily living, which did not reach significance. The main side effects were of reversible nausea and liver function test derangement. No serious adverse events related to the treatment were reported. A randomized controlled trial of adequate duration with nicotinamide is warranted to determine clinical efficacy and long-term safety.

Increased DNA methylation has also been noted in GAA-flanking regions in FRDA lymphoblastoid cells, PBMCs, and buccal cells. The extent of CpG methylation was shown to predict age of onset and levels of FXN expression as well as clinical outcome (39). Antisense transcription has also been implicated in FRDA with increased levels of FXN antisense transcript 1 (*FAST1*) noted in FRDA fibroblasts compared to unaffected individuals. Additionally, siRNA knockdown of *CTCF*, a chromatin insulator protein known to prevent the spread of heterochromatin, upregulated *FAST1* and reduced FXN transcription (40). A recent study that screened 1600 available drug compounds found an improvement in FRDA mouse phenotype and a 1.5-fold increase in frataxin expression in disease-specific tissues following treatment with dyclonine, a topical anesthetic that can cross the blood–brain barrier (41). FRDA subjects given dyclonine as an oral rinse, which is already FDA approved, twice a day for 7 days, resulted in increased frataxin protein in buccal cells. Dyclonine both induces Nrf2 transcription factor that binds an upstream response element [nuclear factor (erythroid-derived 2)-like 2] in the FXN locus as well as inhibiting the histone methyltransferase G9a (PubChem, assay ID 504332), which is known to methylate H3K9, a marker of transcriptional repression (1, 41). Interestingly, the use of another G9a inhibitor (BIX-01294) also showed a trend toward increased frataxin expression to a similar extent in FRDA lymphoblastoid cell lines. However, this was not statistically significant, and it had no effect in another study (15, 42). This partial release from silencing may be in part due to the known presence of both H3K9me3 and H3K27me3 at the *FXN* locus with the possibility of complete release following antagonism of both such marks (13). It should also be noted that carriers with one expanded allele are asymptomatic and only express half the frataxin of unaffected individuals, further supporting the importance of these mechanisms in disease modification (12–14).

## Fragile X Syndrome and Fragile-X-Associated Tremor/Ataxia Syndrome

Another disease caused by triplet-repeat expansion that affects expression of the gene is fragile X syndrome (FXS) that is the most common form of inherited mental retardation with those affected often exhibiting an autistic spectrum disorder. It is an X-linked dominant disease with variable penetrance, affecting 1 in 2500 males and 1 in 4000 females (43). Its name stems from the discontinuation of staining at the Xq27.3 cytogenetic band (i.e., a “fragile site”) when cells are cultured in folate-deficient medium, where the FMR1 gene is located. In addition to cognitive difficulties (often more severe in males due to its presence on only one X chromosome), patients can also exhibit facial dysmorphism and macroorchidism in males. The disorder is the result of a (CGG)n triplet-repeat expansion in the 5′ untranslated region of the fragile X mental retardation 1 (*FMR1)* gene (44). DNA methylation, alteration of the histone code, and toxic RNA gain-of-function have all been implicated in the pathogenesis of FXS (43–52). Those with a full mutation (>200 repeats) were noted in to have hypermethylated CpG sites along the *FMR1* promoter region with subsequent loss of fragile X mental retardation protein (FMRP). FMRP is a RNA binding protein that can bind its own RNA among others, permitting RNA transport along neuronal dendrites and is implicated in synaptic maturation (45). Treatment of FXS cells with the DNA methylation inhibitor 5-aza-2-deoxycitidine was able to reduce levels of CpG methylation and reactivate *FMR1* expression (50). The histone signature was further defined with evidence of H3K9me3 and H4K20me3 in close proximity to the repeat and a broader distribution of H3K9me3 and H3K27me3 (51). The complexity of epigenetic gene regulation is highlighted in the finding of two unrelated males with FXS full CGG expansion, which were atypically found to have unmethylated *FMR1* DNA within the promoter regions. Histone H3/H4 acetylation and H3K9me1 were similar to those of typical FXS cell lines with methylated promoters. H3K4 and H3K27 trimethylation levels were similar to unaffected control individuals with no repression of the FMR1 gene. However mRNA translational efficiency was shown to be reduced (46). Recently, the formation of an RNA–DNA duplex, whereby RNA transcripts directly interact with the FMR1 promoter, has been shown to cause gene silencing (52). Treatment with an inhibitor of linearization (named “1a”) of the RNA hairpin during development of a FXS embryonic stem cell prevented gene silencing by preventing duplex formation (52). Those FXS alleles carrying 55-200 repeats are classed as having a pre-mutation, which has been associated with an increased propensity to develop autistic features or anxiety disorder (43). Twenty percent of female carriers suffer premature ovarian failure. Fragile-X-associated tremor/ataxia syndrome (FXTAS) also develops in pre-mutation carriers, with *FMR1* mRNA increased 2–10 times, with no subsequent increase in FMRP protein, indicating a toxic RNA gain-of-function mechanism behind FXTAS (43, 53). This apparent fine regulation of *FMR1* transcription, with either too little or too much *FMR1* transcript being pathogenic, poses a challenging hurdle for those investigating therapeutic agents aimed at increasing *FMR1* expression.

## Myotonic Dystrophy

Myotonic dystrophy (DM) is an autosomal dominant neuromuscular disorder and the commonest form of adult muscular dystrophy affecting 1 in 8000 within Caucasian populations (54). Characteristic signs are those of myotonia (skeletal muscle hyperexcitability), progressive muscular dystrophy, cataracts, cardiac conduction defects, cognitive deficits, and endocrine anomalies. Myotonic dystrophy type 1 (DM1) is caused by a (CTG)*n* expansion in the 3′ untranslated region of the gene, dystrophia myotonica protein kinase (*DMPK*), on chromosome 19 (54). Expanded CTG repeats are highly unstable in both germline and somatic tissues, with the phenomenon of genetic anticipation being first described in a family with DM1 whereby successive generations have a tendency to exhibit a more severe phenotype than their ancestors (55, 56). The number of repeats is correlated with the severity of symptoms and the earlier disease onset providing a molecular explanation for anticipation. Individuals with 5–37 repeats are unaffected, while >50 repeats results in disease, congenital onset being seen in those with very large expansions (e.g., 1500 repeats) (54–57).

A number of mechanisms have been proposed for the pathological role of CTG repeats in DM (58). Dominantly a spliceopathy is implicated in the pathogenesis, whereby RNA foci formed by triplet-repeat transcripts result in sequestration of muscleblind-like (MBNL) proteins and the upregulation of CUG-binding (CUGBP)/Elav-like family member 1 (CELF1) proteins, possibly through an increase in CUGBP’s half-life (58). These proteins are required for mammalian myoblast differentiation. Importantly, misplacing of a gene that encodes an ion channel CLCN1 occurs in DM1 and direct mutation of this ion channel in another disease causes myotonia (59). Thus, imbalance between levels in regulators of splicing may explain the phenotypic heterogeneity in DM1 (60–62). The CTG repeat has also been shown to influence chromatin, making it less permissive for transcription (63), as is the case for the heterochromatin-mediated silencing triggered by CTG expansions using the hCD2 reporter gene in a similar manner to GAA expansions (25) and in patient-derived fibroblasts, nuclease resistance of the SIX5 enhancer present in the 3′ region of the *DMPK* gene, renders it inaccessible to transcription factors and causing downregulation of SIX5 expression (63–65). Repression of the SIX5 gene has been shown to lead to the development of cataracts in mice, which is a dominant symptom of myotonic dystrophy (66). Repeat instability is key in the pathogenesis of DM1, with muscle tissue showing the greatest incidence of somatic repeat expansion, dictating severity of disease. Increased repeat instability was shown in cells from DM patients treated with DNA methyltransferase inhibitor 5-aza-deoxycytidine (67). Loss of CTCF boundary regions flanking the CTG repeats have been shown to be associated with DNA hypermethylation (68). Activation of DMPK antisense transcription resulted in enrichment of H3K9 methylation and HP1gamma at regions flanking CTG repeats, providing further support for heterochromatic spreading from the CTG repeat region (68). Interestingly, the maintenance of transcription at this heterochromatinized locus may be the result of the loss of CTCF binding with an insulator loss-of-function promoting action of nearby promoters and enhancers. This theory is supported by increased DMPK expression during late embryogenesis in congenital DM1. In adult onset DM1, CpGs in the region of the CTG expansion are unmethylated and DMPK expression is lower; while in congenital DM1, CpGs are aberrantly methylated preventing CTCF binding allowing action of the SIX5 enhancer (69). As CTGs can trigger heterochromatin formation, it is possible that the DMPK gene is in fact causing variegated silencing. To our knowledge, this has not been examined in detail. If this were the case, a potential therapeutic approach would be to increase the silencing with epigenetic modifiers. Interestingly, a current therapeutic approach being investigated is to silence genes using exon-skipping as is the case with Huntington’s disease (HD).

## Huntington’s Disease

Huntington’s disease is an autosomal dominant disease with a prevalence of 10 per 100,000 people (70). It is a progressive neurodegenerative disorder typically presenting in the fourth or fifth decade with generalized chorea. Cognitive impairment and behavioral change are also frequently exhibited, and often precede the movement disorder. Dementia is a late feature of the disease. HD is the result of a heterozygous (CAG)n expansion within exon 1 of the *huntingtin* gene (*HTT*) (71). This results in a polyglutamine expansion within the HTT protein. As with other trinucleotide repeat disorders, there is an apparent repeat threshold, which dictates both likelihood of disease onset as well as subsequent age of onset (72, 73). Those with 6–35 repeats are unaffected, while greater than 40 repeats is causative. Individuals with 36–39 repeats have a variable penetrance and often present late with the disorder. Due to repeat instability, there is an increased risk to children of intermediate length mutation carriers developing the disease (72). Juvenile onset HD is associated with large expansions. The (CAG)n expansion results in misfolding of the HTT protein and following cleavage, intracellular aggregate formation (74, 75). It is the interruption of numerous cell processes by these aggregates, which is implicated, in the neuronal toxicity and cell death in HD (70, 74–78).

Unlike many other TNR disorders, the HD gene locus is not known to be overtly heterochromatinized, allowing for transcription. However, single cell gene expression analysis is lacking and would greatly increase our understanding of the transcriptional kinetics of the pathological *HTT* gene. As TNR in HD are often much shorter than in other TNR diseases, this may explain the lack of compaction at this locus (73). Furthermore, it has been shown that the location of transgenes containing CAG expansions in a heterochromatic environment resulted in reduced somatic instability, whereas those integrated in an open chromatin environment had more instability that may be in part due to the increased accessibility to the transcriptional machinery (77). The possibility of increased somatic instability, which has been implicated in disease progression in HD and DM1, should be considered in studies using agents such as HDAC inhibitors in HD (77).

Hypoacetylation of histones H3 and H4 as well as the discovery of Creb-binding protein (CBP, a histone acetyl transferase and transcriptional activator) within intracellular inclusions was shown in several HD animal models, human cell lines, and post-mortem tissues (78). In support of the association of CBP with the pathogenesis of HD, overexpression of CBP reversed neuronal toxicity in cells expressing mutant HTT (78). Further evidence for the importance of histone and non-histone protein acetylation in HD has been shown through the effect of HDAC inhibitors (HDACi) on several HD models (79–83). Neuronal degeneration was shown to be reduced in Drosophila models of HD following treatment with HDACi. Reduction of RPd3 (ortholog to HDAC1/2/8 in humans) or Sir2 (Sirt1 in humans) was also neuroprotective in another study (80).

Increased transcription of alpha thalassemia/mental retardation X linked (ATRX) protein has also been found in HD models (84). ATRX is an ATPase/helicase that binds to the repressive mark H3K9me2/3 and co-localizes with HP1-alpha (1, 85). ATRX overexpression increased H3K9me3 and pericentromeric heterochromatin condensation as well as eye degeneration in a HD fly model (84–86). H3K4me3 was reduced at transcriptionally repressed promoters in HD mice and patients (87). This is thought to be caused by an increase in KDM5C, a histone lysine demethylase, which was protective in HD mouse and *Drosophila* models (87).

DNA methylation has also been implicated in epigenetic dysregulation in HD (70, 79, 88). Comparison of genome-wide CpG methylation status between control cells and those expressing mutant HTT found that a significant number of downregulated genes in HTT cells were preferentially methylated (79). Two frequently downregulated genes are brain-derived neurotrophic factor (BDNF) (important for mature neuron survival) and the adenosine A2A receptor (79, 89, 90).

## Spinocerebellar Ataxias

Spinocerebellar ataxias (SCAs) are a group of dominantly inherited neurodegenerative disorders that predominately affect the cerebellum, brainstem, and spinal cord. Several SCAs are the result of polyglutamine (CAG) repeat expansions in the coding region of genes (SCA-1, 2, 3, 6, 7, and 12). Epigenetic gene regulation mechanisms have been implicated in the pathogenesis of several SCAs (91–96).

SCA7 is the result of an expansion in the N-terminal region of the ATXN7 gene, patients exhibit ataxia as well as retinal dysfunction (91). ATXN7 is a subunit of the SAGA complex, a multi-subunit complex that can acetylate and deubiquitinate histones and other non-histone substrates (92). There is conflicting information regarding the effect of the expanded tract on the histone acetyl transferase activity of the SAGA complex. One study in SCA7 mice found increased acetylation of H3K9 and H3K14 at rod cell-specific gene promoters, surprisingly this was associated with a reduction in mRNA (92, 93). Other studies in SCA7 yeast models and mammalian cell lines showed a reduction in H3 acetylation through reduced acetyltransferase activity (94).

SCA8 is the result of a CAG repeat expansion in the ataxin 8 gene and a CTG expansion at the 3′UTR of the antisense strand (95). An increase in H3K9me2 and reduction in H3K14ac were noted in ATXN8OS cells lines with 157 repeats, but not in those with 23 or 88 repeats (97). Similar to findings in DM1, a toxic RNA gain-of-function mechanism has been implicated with splicing changes and increased expression of the CUGBP1–MBNL1 regulated CNS target, GABA-A transporter 4 (96).

A similar toxic RNA gain-of-function mechanism has been implicated in the pathogenesis of SCA3 whereby experiments in Drosophila showed disruption of CAG repeats by insertion of a CAACAG sequence that was able to alleviate neurodegeneration, while expression of the untranslated CAG repeat caused neurodegeneration (98).

## Spinal and Bulbar Muscular Atrophy

Spinal and bulbar muscular atrophy (SBMA) is an X-linked recessive disorder and the first TNR disorder described. It is caused by a pathogenic CAG expansion in the first exon of the androgen receptor. Males are affected and exhibit motor neuron degeneration in the spinal cord and brainstem and wasting of limb muscles. There is also subclinical loss of sensory neurons in the dorsal root ganglia. HDAC inhibitors have been shown to effectively treat the disease phenotype and correct histopathological abnormalities in a mouse model of SBMA (97).

## Conclusion and Future Directions

It is evident that epigenetic gene regulation is integral to the pathogenesis of several trinucleotide repeat diseases, with mutations resulting in DNA methylation, local heterochromatinization, RNA loss- or gain-of-function, aberrant mRNA splicing, antisense transcription, and/or protein aggregation being predominant mechanisms. Furthermore, global alterations in gene expression are implicated in the varied disease course and phenotype of many TNR disorders. This epigenetic layer of gene regulation brings with it the prospect of new therapeutic targets. Global activation or inhibition of these highly conserved epigenetic targets is likely to result in off-target effects, posing the need for further work in improving specificity of interventions for each disease. The answers to key questions on mechanisms of epigenetic regulation will bring us closer to more effective and selective therapies for these diseases.

## Glossary

**R-loop:** A loop of single-stranded DNA formed when mRNA binds to its complementary exonic regions of double-stranded DNA. Unbound intronic DNA forms R-loops.

**CTCF:** Eleven zinc-finger protein that binds in a sequence-specific manner to DNA. Regulates 3D structure of the genome by forming chromatin loops and forms boundary regions between euchromatin and heterochromatin.

**Heterochromatin:** Regions of compact chromatin, mainly consisting of transcriptionally inert satellite sequences. Classically found at centromeres and telomeres.

**Euchromatin:** Open chromatin, providing an environment to permit gene expression.

**Repeat instability:** The propensity for repetitive sequences of DNA to undergo expansion and contraction.

**Anticipation:** The onset of symptoms of a genetic disorder at an earlier age in subsequent generations, often due to expansion in the case of triplet-repeat disorders. More frequently seen through paternal inheritance.

**Histone deacetylase (HDAC):** Enzyme that promotes the removal of acetylation from histone and other non-histone proteins. Permitting addition of methylation.

## Conflict of Interest Statement

The authors declare that the research was conducted in the absence of any commercial or financial relationships that could be construed as a potential conflict of interest.
